# Chromosome-level genome assembly of an oligophagous leaf beetle *Ophraella communa* (Coleoptera: Chrysomelidae)

**DOI:** 10.1038/s41597-024-03486-8

**Published:** 2024-07-06

**Authors:** Yi-Ting Wang, Yan Zhang, Chao Ma, Wei-Hua Ma, Li-Jun Cao, Jin-Cui Chen, Wei Song, Jing-Fang Yang, Xu-Yuan Gao, Hong-Song Chen, Zhen-Ya Tian, Nicolas Desneux, Shu-Jun Wei, Zhong-Shi Zhou

**Affiliations:** 1 State Key Laboratory for Biology of Plant Diseases and Insect Pests, Institute of Plant Protection, Chinese Academy of Agricultural Sciences, Beijing, 100193 China; 2 Institute of Plant Protection, Beijing Academy of Agriculture and Forestry Sciences, Beijing, 100097 China; 3 National Nanfan Research Institute, Chinese Academy of Agricultural Sciences, Sanya, 572019 China; 4 Hubei Insect Resources Utilization and Sustainable Pest Management Key Laboratory, College of Plant Science and Technology, Huazhong Agricultural University, Wuhan, 430070 China; 5 Guangxi Key Laboratory for Biology of Crop Diseases and Insect Pests, Institute of Plant Protection, Guangxi Academy of Agricultural Sciences, Nanning, 530007 China; 6 French National Institute for Agriculture, Food, and Environment, Nice, 06000 France

**Keywords:** Agricultural genetics, Genome

## Abstract

The leaf beetle *Ophraella communa* LeSage (Coleoptera: Chrysomelidae) is an effective biological control agent of the common ragweed. Here, we assembled a chromosome-level genome of the *O. communa* by combining Illumina, Nanopore, and Hi-C sequencing technologies. The genome size of the final genome assembly is 733.1 Mb, encompassing 17 chromosomes, with an improved contig N50 of 7.05 Mb compared to the original version. Genome annotation reveals 25,873 protein-coding genes, with functional annotations available for 22,084 genes (85.35%). Non-coding sequence annotation identified 204 rRNAs, 626 tRNAs, and 1791 small RNAs. Repetitive elements occupy 414.41 Mb, constituting 57.76% of the genome. This high-quality genome is fundamental for advancing biological control strategies employing *O. communa*.

## Background & Summary

The leaf beetle *Ophraella communa* LeSage (Coleoptera: Chrysomelidae) is a native to North America^1^. It has been identified as a biological control agent of the common ragweed, *Ambrosia artemisiifolia*, a harmful invasive weed. It has achieved great success in controlling *A. artemisiifolia* spread and damage in different regions worldwide^2–4^. *O. communa* is an oligophagous insect feeding on various plants of the *Asteraceae* family and poses no threat to commercial crops. This beetle has short developmental periods, high fertility, and a long lifetime. The larvae and adults of *O. communa* can completely defoliate a common ragweed within a few insect generations^5^. To better apply this beetle, studies on its chemical ecology^6–8^, reproductive biology^9–11^, and cold tolerance genetics^12^ have been ongoing. Bouchemousse, *et al*.^13^ assembled a draft genome for *O. communa* on scaffold level^14^. A high-quality assembled and annotated genome is essential to assess potential adaptive processes by identifying underlying genetic mechanisms.

To this end, we applied Nanopore long-read, Illumina short-read sequencing, and High-throughput chromosome conformation capture technologies (Hi-C) to generate the first chromosome-level genome of *O. communa*. The assembled genome consists of a total scaffold length of 733.1 Mb, mapping to 17 chromosomes. Compared to the published contig version^14^, the contig N50 increased from 195.5 Kb to 7.05 Mb. A total of 414.41 Mb repeat sequences representing 57.76% of the whole genome were identified. Among these repeat sequences, 15.51% were classified as known repeat elements. We then performed structural and functional annotation on the obtained genome, incorporating transcriptome data from all developmental stages of *O. communa*. As the first chromosome-level genome assembly in the genus *Ophraella*, this high-quality reference genome not only provides information for better improvement of the biological control potential of *O. communa*, but also serves as a valuable resource for understanding the genetics, ecology, and evolution of *Ophraella* beetles.

## Methods

### Sample preparation and genomic DNA sequencing

A population of the *O. communa* collected from Guangxi, China, was established in the laboratory at the Institute of Plant Protection, Chinese Academy of Agricultural Science. This inbred population was fed with common ragweed for approximately ten generations in the laboratory under the following conditions: temperature of 27 ± 1 °C, relative humidity of 70 ± 5%, and a photoperiod of 14 L:10D. All the samples used in this study were from this inbred population which shared the almost same genetic background. Due to the small size of the *O. communa* pupa and its high fat content, the extracted DNA from one pupa is insufficient to conduct multiple sequencing methods. Thus, one pupa was used for the Nanopore library and another for Illumina library construction. The genomic DNA was extracted using the CTAB method. After removing the pupal shell, epidermis, and extracting as much fat body tissue as possible, the remaining tissue was homogenized in CTAB extraction buffer (20 g/L CTAB; 1.4 mol/L NaCl; 0.1 mol/L Tris-HCl; 20 mmol/L Na_2_EDTA). Then, the genomic DNA was purified using a Blood and Cell Culture DNA Midi Kit (QIAGEN, Germany). The purity of the extracted DNA was assessed through 0.75% agarose gel electrophoresis, while the concentration was assessed using a Qubit 2.0 Fluorometer (Thermo Fisher Scientific, USA). Illumina paired-end (PE) library featuring an insert size of approximately 350 bp utilized the TruSeq Nano DNA HT Sample Preparation Kit (Illumina, San Diego, California, USA). Subsequently, paired-end reads of 150 bp were generated on the Illumina NovaSeq 6000 platform. The output amounted to 83.00 GB of clean data, providing coverage of 115.67 × . The G + C content was measured to be 33.32%, and the peak insert size observed was 169 bp. After filtering using fastp version 0.23.4^15^, 82.13 GB of data remained, with 76.63 GB (93.08%) of high-quality sequences exceeding Q30 threshold (Table 1). The long-insert library was constructed with the SQK-LSK108 1D Ligation Sequencing Kit (Oxford Nanopore Technologies, Kidlington, Oxford, UK) using the genomic DNA. The long-insert library underwent sequencing on the Nanopore PromethION sequencer at GrandOmics (Wuhan, China). A total of 105.86 Gb (147.54 × coverage) of long-reads were generated, with a mean Q score of 11.50. The N50 length of clean data is 274,07 bp, the longest reads is 465963 bp, and the average length is 16,445 bp (Table 1).

**Table 1:** Library sequencing data and methods used in this study to assemble the *Ophraella communa* genome.

Sequencing strategy	Platform	Usage	Insertion size	Clean data (Gb)	Coverage (X)
Short-reads	Illumina	Genome survey	350 bp	83.00	115.67
Long-reads	Nanopore	Assembly	12–20 kb	105.86	147.54
Hi-C	Illumina	Hi-C assembly	350 bp	42.56	59.33
RNA-seq	Illumina	Annotation	350 bp	115.13	157.04

### Hi-C library preparation and sequencing

One pupa with unknown sex was used to create the Hi-C library to capture genome-wide chromatin interactions. After washing the surface with PBS buffer, the pupal shell, epidermis and as much fat body tissue as possible were removed. Subsequently, cross-linking was performed using a 2% formaldehyde isolation buffer, followed by treatment with the restriction enzyme *MboI* to digest chromatin. The Hi-C samples were then extracted via biotin labeling and flat-end ligation, after which the ligated DNA was fragmented into 350 bp fragments. Subsequently, the Hi-C library was sequenced on the Illumina NovaSeq platform with paired-end 150-bp reads. The Hi-C Illumina sequencing generated 42.56 Gb (59.33 × coverage) of clean data (Table 1). The G + C content was measured to be 33.50%, and the mean length of reads was 100 bp. After filtering using fastp version 0.23.4^15^, 42.48 GB of data remained, with 40.95 GB (96.39%) of high-quality sequences exceeding Q30 threshold.

### Transcriptome sequencing

To obtain comprehensive genome annotation, we conducted transcriptome sequencing on different stages of *O. communa*, including two adult pooled samples, two egg pooled samples, two pupa pooled samples, and three larva pooled samples. Each adult pooled sample consisted of five male and female adults of *O. communa*. Each egg sample consisted of approximately 100 eggs. Each pupa and larvae samples consisted of five pupae and larvae, respectively. The total RNA was extracted with the TRIzol reagent (Thermo Fisher Scientific, USA). Except for the pupae after removing the pupal shell, all other samples are homogenized using the entire organism with TRIzol. cDNA was synthesized from total RNA using PrimeScript™ RT reagent kit with gDNA Eraser (Perfect Real Time; Takara, Japan) following the manufacturer’s instructions. Then the cDNA was constructed to the paired-end libraries using the VAHTSTM mRNA-seq v2 Library Prep Kit (Vazyme, Nanjing, China) with an insert size of approximately 350 bp and then sequenced on the Illumina NovaSeq 6000 platform with paired-end reads of 150 bp. The nine libraries generated 115.13 GB of clean data, providing coverage of 157.04 × . The mean G + C content was measured to be 38.82%, and the mean peak insert size observed was 265 bp. After filtering using fastp version 0.23.4^15^, 114.47 GB of data remained, with 96.89 GB (93.64%) of high-quality sequences exceeding Q30 threshold (Table 1).

### Estimation of genomic characteristics

**Fig. 1:**
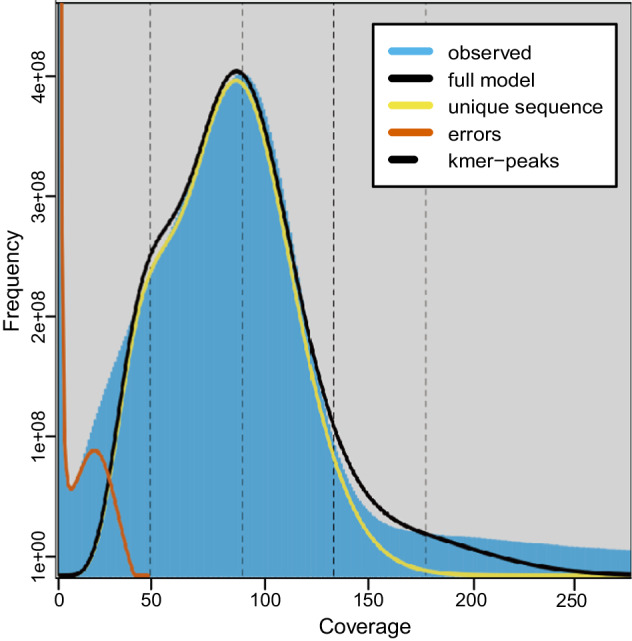
The estimated characteristics of *Ophraella communa* genome based on Illumina short-read data using 25-mers count histogram. Genome size was estimated to be 741.69 Mb, with a duplication rate of 4.9% and heterozygosity rate of 0.73%.

The genome features of *O. communa* were surveyed using the k-mer method based on Illumina short reads. The k-mer count histogram was generated using Jellyfish version 2.2.10 with the following parameters: ‘count -m 25 -C -s 5 G’^16^. We used GenomeScope^17^ version 1.0 to estimate the genome size, heterozygosity, and duplication rate. The analysis based on 25-mers estimated the genome size of *O. communa* to be approximately 741.69 Mb, showing a high degree of duplication (4.9%) and heterozygosity (0.73%) (Fig. 1).

### Genome assembly

The Nanopore long reads were corrected and assembled into contigs using NextDenovo version 2.5.0 (https://github.com/Nextomics/NextDenovo) with parameters: ‘read_cutoff = 1k, genome_size = 750 m, pa_correction = 20, nextgraph_options = -a 1’. Subsequently, the contigs assembly underwent three rounds of polishing using NextPolish version 1.4.0 18^18^, incorporating the parameters: ‘genome_size = auto, sgs_options = -max_depth 100 -bwa, rerun = 3’. To generate a high-quality chromosome-scale genome, the Hi-C data were initially mapped to the contig assembly using BWA-MEM version 0.7.17^19^ with the parameters:‘mem -SP5M’. Then, the *MboI* sites were generated using the’generate_site_positions.py’script in the Juicer^20^ version 1.6 with default parameters. 3D-DNA (3D de novo assembly, version 180114) pipeline^21,22^ was employed to order, orient, and cluster the contigs into scaffolds with a modified parameter of ‘–editor-repeat-coverage 5, -r 2’. To achieve the final chromosome assembly, manual scaffold ordering was performed using Juicebox version 1.11.08 (https://github.com/aidenlab/Juicebox).

**Fig. 2:**
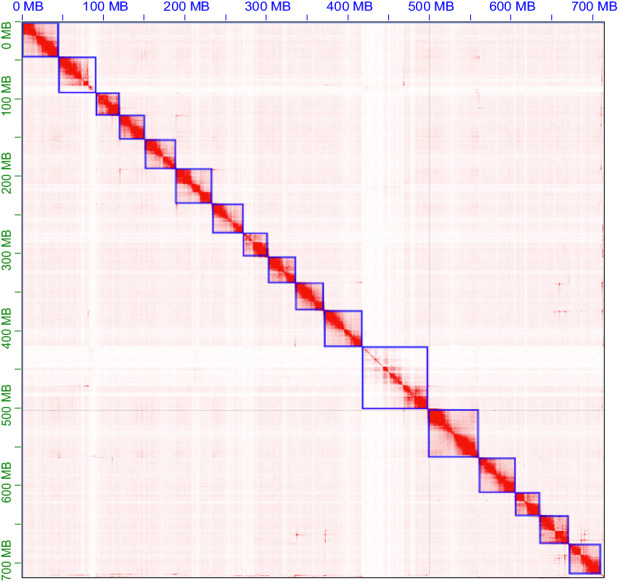
Genome-wide all-by-all Hi-C interaction identified 17 pseudo-chromosome linkage groups of *Ophraella communa*.

At the contig level, the genome is 735.31 Mb, comprising 220 contigs, with a contig N50 of 7.05 Mb (Table 2). At the chromosomal level, the resulting genome of *O. communa* measures 733.13 Mb, organized into 17 scaffold groups, with a scaffold N50 of 45.03 Mb (Table 2). The karyotype of *O. communa* is 2n = 18, consisting of one pair of heteromorphic sex chromosomes (XX in females, XY in males) and 17 pairs of autosomes^13^, which were well-distinguished from the chromatin interaction heatmap (Fig. 2). The scaffold groups vary in length, with the longest group spanning 81.99 Mb and the shortest group spanning 29.41 Mb. The *O. communa* genome has a G + C content of approximately 31.98% (Table 2).

**Table 2:** Features of *Ophraella communa* genome.

Genome feature	Value (bp)
Total length (bp)	733,128,312
Contigs N50 (bp)	7,051,505
Scaffold N50 (bp)	45,027,602
Longest scaffold length (bp)	81,994,011
G + C (%)	31.98
Anchored to chromosome (Mb, %)	717,507,083 (97.87%)

### Repeat element and non-coding RNA annotation

The assembled genome was analyzed for repetitive elements and transposable element families using RepeatMasker version 4.0.7^23^ against the Insecta repeats within RepBase Update (http://www.girinst.org) with parameters of ‘-e ABBlast, -species Insecta’. Additionally, ab *initio* prediction was conducted using the program RepeatModeler version open-1.0.8 (https://www.repeatmasker.org/RepeatModeler/). Non-coding RNAs (ncRNAs) were annotated by aligning the genomic sequence against RFAM (http://rfam.xfam.org/) using version 1.1.2^24^ with the parameter of ‘-e ABBlast’. The prediction of transfer RNA (tRNA) was conducted through tRNAscanSE v.1.3.1^25^ with default parameters. For ribosomal RNA (rRNA) prediction, RNAmmer-1.2^26^ was employed with parameters: ‘-S euk, -multi’.

The repetitive elements constituted 414.41 Mb (57.76%) of the *O. communa* genome, with 15.51% being classified as known repeat elements (Table 3). According to the Rfam databases, our predictions for the *O. communa* genome revealed 204 rRNAs, 626 tRNAs, and 1791 small RNAs (Table 4).

**Table 3:** Repeats elements statistics in genome of *Ophraella communa*.

Items	Number of elements	Length occupied (bp)	Percentage of sequence (%)
Retroelements	139619	57013504	7.95
SINEs	198	12213	0.00
LINEs	107673	31868392	4.44
LTR elements	31748	25132899	3.50
DNA transposons	129099	39916980	5.56
Total interspersed repeats	NA	97628410	13.61
Small RNA	1791	418346	0.06
Satellites	13	2034	0.00
Simple repeats	192320	11323958	1.58
Low complexity	52094	2692621	0.38

Note: SINEs, short interspersed nuclear elements; LINEs, long interspersed nuclear elements; LTR, long terminal repeat.

**Table 4:** Statistics of non-coding RNAs in *Ophraella communa* genome.

Class	Type	Number
rRNA counts	8s_rRNA	94
	18S_rRNA	55
	28s_rRNA	55
	Candidate tRNAs read	435
Cove stats	Cove-confirmed tRNAs	626
	Bases scanned by Cove	556066
tRNA Count	tRNAs decoding Standard 20 AA	348
	Selenocysteine tRNAs (TCA)	0
	Possible suppressor tRNAs (CTA, TTA)	0
	tRNAs with undetermined/unknown isotypes	4
	Predicted pseudogenes	274
Total tRNAs		626
tRNAs with intron		27

### Gene and functional predictions

Protein-coding genes in the were annotated utilizing homolog-based, RNA-seq-based, and ab initio methods using Maker genome annotation pipeline version 3.01.04^27^. The transcriptome of *O. communa* was initially assembled by employing StringTie version 1.3.3b^28^ and PASA version 2.0.2^29^. This process utilized the FASTA files of the final chromosome assembly and transcriptome sequencing reads as input data, with default parameters. Ab initio prediction models were trained using homologous genes from *Tribolium castaneum*^30^ and the transcripts for Augustus version 3.4.0^31^ with default parameters and SNAP version 2006-07-28^32^ with the parameters of ‘-categorize 1000, -export 1000, -plus’. The results were utilized for subsequent rounds of model training and annotation. Three rounds of Maker annotations were conducted and improved by PASA. Then, this result was integrated with the result of a deep-learning structural gene annotations approach Helixer^33^ and then filtered based on gene expression evidence and functional annotation. In order to ensure the accuracy of the annotation results, genes with fragments per kilobase per million (FPKM) values equal to 0 were excluded for further analysis. The protein-coding genes, Gene Ontology (GO), and Kyoto Encyclopedia of Genes and Genomes (KEGG) items underwent annotation using eggNOG-Mapper version 2.1.9 within the Expected eggNOG DB version 5.0.2^34^. This process utilized specific parameters, including ‘–tax_scope auto’, ‘–go_evidence experimental’, ‘–target_orthologs all’, ‘–seed_ortholog_evalue 0.001’, ‘–seed_ortholog_score 60’, and ‘–override’. In the chromosome-level assembly, we annotated 25,873 protein-coding genes, which is closer to the number of genes found in related Coleoptera species and general insect genomes compared to the 75,642 protein-coding genes in the genome reported by Bouchemousse *et al*.^13^. In total, 25,873 protein-coding genes were annotated, with 22,084 genes (85.35%) being functionally annotated^35^.

## Data Records

The *O. communa* genome project was deposited at NCBI under the BioProject accession number PRJNA899605. Genomic Illumina sequencing data are available in the Sequence Read Archive at NCBI under accession SRR27238374^36^. Hi-C sequencing data are available in the Sequence Read Archive at NCBI under accession number SRR27307846^37^. Genomic Nanopore sequencing data are available in the Sequence Read Archive at NCBI under accession number SRR27290278^38^. RNA-seq data are available in the Sequence Read Archive at NCBI under accession number SRR27334077-SRR27334085^39–47^. The final chromosome assembly was deposited in GenBank at NCBI under accession number GCA_035357415.1^48^. The genome annotation files are available in Figshare under a DOI of 10.6084/m9.figshare.24901596.v1^35^.

## Technical Validation

The accuracy of the final genome assembly was assessed by aligning Illumina short reads and RNA-seq data to the *O. communa* genome using BWA-MEM2 version 2.2.1 (https://github.com/lh3/bwa). The analysis revealed that mapping rate of 99.56% for the short reads to the genome. The mapping rates for the respective stages-specific transcriptomic data ranged from 89.39% to 94.12%.

In evaluating the completeness of the *O. communa* genome, an analysis was conducted using BUSCO version 5.2.2^49^ with the insecta-odb10 database, which consists of 1,367 genes. The BUSCO analysis revealed that 99.7% of the evaluated single-copy genes at the contig level were determined to be complete (96.6% single-copy genes and 3.1% duplicated genes). At the chromosome level, it was observed that 99.7% of the assessed single-copy genes were classified as complete (97.1% single-copy genes and 2.6% duplicated genes). For all protein-coding genes and functionally annotated protein-coding genes, it was determined that 95.1% of them were identified as complete (92.7% single-copy genes and 2.4% duplicated genes) (Table 5).

**Table 5:** Completeness of the assembled genomes and sets of protein-coding genes evaluated by BUSCO analysis.

Data	Complete gene%	Single-copied gene%	Duplicated gene %	Fragmented gene%	Missing gene%
Contig level assembly	99.7	96.6	3.1	0.1	0.2
Chromosome level assembly	99.7	97.1	2.6	0.1	0.2
All protein-coding gene	95.1	92.7	2.4	2.5	2.4
Functionally annotated protein-coding gene	95.1	92.7	2.4	2.5	2.4

## Data Availability

No custom scripts or code were used in this study.
